# Appendicular Actinomycosis: Behind the Curtains of Appendicitis

**DOI:** 10.7759/cureus.29709

**Published:** 2022-09-28

**Authors:** Sara Completo, Marta Veríssimo, André M. G. Pereira, Isabel França, Piedade Sande Lemos

**Affiliations:** 1 Pediatric Service, Child and Youth Department, Hospital Prof. Doutor Fernando Fonseca, EPE, Amadora, PRT; 2 Anatomopathology Service, Hospital Prof. Doutor Fernando Fonseca, EPE, Amadora, PRT; 3 Pediatric Surgery Service, Child and Youth Department, Hospital Prof. Doutor Fernando Fonseca, EPE, Amadora, PRT

**Keywords:** chronic granulomatous disease, inflammatory bowel disease, recurrent abdominal pain, acute appendicitis, actinomyces infection, visceral actinomycosis

## Abstract

Actinomycosis is a rare, indolent, and multisystemic infection caused by Actinomyces, commensal bacteria of the oral and intestinal flora. It usually occurs due to tissue disruption. It affects the abdominal region in 20% of cases, and the most common presentation is a perforated appendix. Symptoms are nonspecific, which makes differential diagnosis a challenge.

We present the case of a healthy, nine-year-old boy of African ancestry with a five-month history of recurrent intermittent abdominal pain in the periumbilical and right lower quadrant areas. He recurred to the emergency department with symptoms suggestive of acute appendicitis and was submitted to an emergent laparotomy. The histologic examination revealed *Actinomyces* colonies compatible with the diagnosis of appendicular actinomycosis. He was treated with intravenous penicillin for a month and, subsequently, with oral amoxicillin for a year. He had complete remission of symptoms.

Actinomycosis is a rare entity, particularly in children. Nevertheless, it should be considered in the differential diagnosis of an intrabdominal mass or unspecific recurrent, indolent, and abdominal pain. As symptoms are nonspecific, it can mimic other diseases. It is mostly diagnosed post-operatively, after histological examination. Early treatment is important to avoid recurrence, and, therefore, a high index of suspicion is required.

## Introduction

Actinomycosis is a rare, heterogeneous, chronic granulomatous disease caused by filamentous, gram-positive anaerobic bacteria *Actinomyces*, which are part of the commensal flora of the oral and intestinal mucosa [1-4]. This infection usually occurs after damage of tissues by contiguous growth. It affects predominantly in the cervicofacial region, followed by abdominal and thoracic regions [4-6]. Perforated appendicitis is the most frequent presentation of abdominal actinomycosis [7].

Symptoms are nonspecific, and they may mimic other more frequent diseases, such as appendicitis, inflammatory bowel disease, malignancy or tuberculosis. For this reason, diagnosis might be difficult [1-2], and it is usually reached after surgery, during histological examination, when *Actinomyces* colonies can be detected [2-4,6].

We present a case of a possible course of appendicular actinomycosis in a previously healthy child.

## Case presentation

A nine-year-old boy of African ancestry, born in Portugal but resident in Angola until two years before admission, with no relevant medical background aside from dental cavities.

He had a five-month history of recurrent, intermittent, moderate abdominal pain (with a maximum numeric pain rating of 5-6/10) located in the periumbilical area and right lower quadrant, with no irradiation. There was no vomiting, diarrhea, bloody stools, fever, weight loss or other symptoms. There was a transitory relief of the pain after bowel movement, with no worsening factors. He did an ultrasonography which showed signs suggestive of inflammatory bowel disease, reason why he was first referred to a pediatric gastroenterology appointment. While under gastroenterology investigation, before starting treatment, the child presented at the emergency department due to increasing pain in the right lower quadrant. During examination, he showed mild tenderness and a painful abdomen in the right lower quadrant, with no signs of peritoneal irritation; Signs of McBurney, Rovsing, Psoas and Obturator were negative, and there wasn’t any involuntary guarding or rebound tenderness. Laboratory examination showed slightly low hemoglobin 10.1 g/dL (11.5-15.5 g/dL), leukocytosis 17800/uL (4.5 - 13.5x109/L), neutrophilia 16600/uL (1800-6900/uL) and increased Reactive C Protein (RCP) of 6.5 mg/dL (<0.5 mg/dL).

**Figure 1:**
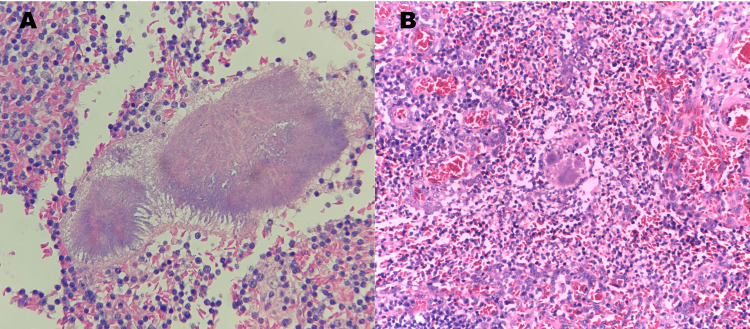
Histological Examination High power view of a histological preparation stained with Hematoxylin and Eosin. It is possible to see the irregular round *Actinomycetes* colonies rimmed by eosinophilic, club-like projections of proteinaceous material (Splendore-Hoeppli material) (A), admixed in the markedly inflamed lamina propria (B).

An abdominal ultrasonography was performed, with sonological findings suggestive of an inflamed appendix, compatible with uncomplicated acute appendicitis. Therefore, he underwent an emergent laparotomy. During this procedure, a large mass comprising a thickened cecum and appendix was identified, excised, and sent to pathology. The histological examination revealed a moderate transmural lymphoplasmacytic inflammatory infiltrate with eosinophilic and lymphoid follicles; the muscular wall was thickened with fibrosis and neural hyperplasia. *Actinomyces* colonies were identified in the lumen of the appendix and on its wall using Hematoxylin and Eosin staining (Figure 1), which led to the diagnosis of appendicular actinomycosis.

The child was admitted to the pediatric inward and was medicated with intravenous penicillin for one month. During hospitalization, etiological investigation of actinomycosis was performed. Blood cultures were negative and immunodeficiencies were excluded, with negative human immunodeficiency virus (HIV) serology, normal immunoglobulins, lymphocyte populations, phagocytosis and oxidation tests and activity of the complement system. Also, inflammatory bowel disease had to be excluded. In that context, anti-saccharomyces cerevisiae antibodies (ASCAa) and antineutrophil cytoplasmic antibodies, perinuclear pattern (pANCA), were negative. Fecal calprotectin was positive (104 mg/Kg). No other risk factors could be identified aside from dental cavities.

He had an excellent evolution, with a resolution of symptoms shortly after surgical removal of the appendix. He was dismissed from the hospital after a month of endovenous therapy and was subsequently treated as an outpatient, with oral amoxicillin, for 12 months.

He had infectious diseases appointments every two months after dismissal for two years. During this time of follow-up, the child remained asymptomatic, and the abdominal ultrasonography after six months of surgery did not show signs of bowel inflammation or intraperitoneal collections.

## Discussion

Actinomycosis is a rare infection caused by *Actinomyces*, gram-positive, anaerobic bacteria that are part of the normal commensal flora of the oral and intestinal mucosa [1-4]. It affects predominantly middle-aged individuals [4-6] and is even more infrequent in pediatric patients, representing only 3% of all infections [6]. It is difficult to ascertain the absolute number of this disease; nonetheless, it is estimated to have an incidence of 0.3-1 episode per year per 100000 [3,5], and its global incidence is decreasing [8]. It causes appendicitis in only 0.02%-0.06% of the cases [4]. It is more frequent in males (1:1,5), but an increasing prevalence in women due to intrauterine contraceptive devices has been described [4,8]. It is more prevalent in developing countries and rural areas, where healthcare systems are poorer [1]. Risk factors, such as immunosuppression, are still unclear and need further studies [1,2].

Actinomycosis usually occurs after the disruption of tissues. It affects most frequently the following regions: cervicofacial (50%), abdominal (20%), and thoracic (15-20%) [4-6]. In abdominal actinomycosis, 65% of the cases affect the appendix, ileum, and cecum [3-5]. Perforated appendicitis is the most frequent cause of this subtype [8]. It can also be related to abdominal trauma, recent abdominal surgery, diverticulitis, abdominal perforation, neoplasia [3-6], or the use of intrauterine contraceptive devices [3,6,7]. There are also reported cases associated with poor dental hygiene [1,3].

Symptoms are nonspecific; therefore, confusion with other more frequent diseases, such as appendicitis, inflammatory bowel disease, malignancy, or tuberculosis, may occur [1,2]. They include abdominal pain associated with a palpable mass, the latter more frequent in children [6], as well as fatigue, fever, weight loss, and constipation [3,5]. In one-third of the children, fistulas and sinus tracts can be identified [6]. In our patient, the initial presentation was recurrent abdominal pain and a thickened cecum, being inflammatory bowel disease the first clinical suspicion. Later on, the increase in abdominal pain and tenderness in the right lower quadrant led to the suspicion of acute appendicitis, which is common in this situation.

In around 90% of the cases, actinomycosis is diagnosed post-operatively by anatomopathological analysis [2-4,6]. It may present with a large, solitary mass, either with or without mucosal ulceration. The inflammatory process often infiltrates surrounding structures, mimicking malignancy, and may produce abscesses or sinus tracts where sometimes the *Actinomyces* colonies are seen as small yellow, "sulfur" granules. Microscopically, this organism typically produces irregular round clusters of bacteria rimmed by eosinophilic, club-like projections of proteinaceous material (Splendore-Hoeppli material). Gram stain reveals the filamentous, gram-positive organisms. In the appendix, transmural inflammation, lymphoid hyperplasia, and marked fibrosis are common histologic features, along with mucosal ulceration and architectural distortion [3,5,7,8].

As microbiological exams are rarely positive, the real incidence of abdominal actinomycosis is underestimated [5,6]. Although none of the image findings are specific, abdominal computerized tomography (CT) [1,3] and magnetic resonance imaging (MRI) are the most useful [3].

Medical treatment consists of intravenous penicillin (25-30mg/Kg) every 4-6 hours for 4-6 weeks, followed by oral penicillin or an equivalent such as amoxicillin for 6-12 months [6-8]. Alternatives include erythromycin, clindamycin, doxycycline, or tetracyclines [3]. The duration of the treatment is still controversial. However, more recently, shorter courses of antibiotics have been established with effectiveness [2]. The combined therapy of surgery and antibiotic appears to cure more than 90% of actinomycosis [8], having an excellent outcome in avoiding recurrence, especially when the infection is treated in an early stage [2,8].

The case we present was diagnosed by anatomopathological findings after surgery of uncomplicated appendicitis and treated with intravenous penicillin for a month and subsequently with oral amoxicillin for one year. This is consistent with the remaining literature [3,4,5,7], where only one paper is related to a pediatric patient [5].

## Conclusions

We report this case due to the rarity of this entity, highlighting the importance of considering actinomycosis in the differential diagnosis of an intrabdominal mass or unspecific, recurrent abdominal pain, especially if it has an insidious, chronic evolution. Symptoms are unspecific, making the diagnosis a challenge, so a high index of suspicion is needed to diagnose these patients. It is mostly diagnosed post-operatively by anatomopathological analysis. Its incidence is underestimated due to a lack of positive microbiological exams. Combined medical and surgical therapy is efficient in preventing recurrence, particularly if initiated in an early stage. If actinomycosis is uncomplicated, antimicrobial therapy might be sufficient. Therefore, it is crucial to obtain an early diagnosis, establish adequate treatment and contribute to prompt resolution of the disease and prevention of recurrence.
